# Comment on “Sexism in Academia is Bad for Science and a Waste of Public Funding”

**DOI:** 10.1002/gch2.202400072

**Published:** 2024-04-24

**Authors:** Leonie Barner

**Affiliations:** ^1^ Centre for a Waste‐Free World School of Chemistry and Physics Faculty of Science Queensland University of Technology 2 George St Brisbane Queensland 4000 Australia

**Keywords:** accountability, bias, funding, gender equity, sexism, STEM, transparency

## Abstract

A recent comment by Boivin et al. urges academia and governments to address sexism and fight bias at higher education and research institutions as losing female academics is costing science and society too much. Herein, I discuss further underlying reasons of sexism in academia and the importance of a deep dive into the causes of inequity at individual faculty and school levels to develop bespoke and enforceable gender equity plans, the importance of not using basic statistic as the only tool to measure equity/inequity as well as how key performance indicators could be better used to advance gender equity and end sexism in academia.

## Graphical abstract

Many early, mid‐, and advanced career female academics face sexism at their institutions as highlighted by a recent comment from Boivin et al. The current comment discusses additional underlying causes of sexism in academia and additional tools to improve equity in academia. Ending sexism in academia is a true global challenge.

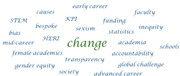

## 1 Introduction

The recent comment from Boivin et al. urges academia to address sexism at their institutions as the cost of losing women at early as well as advanced career stages is too high for themselves as well as society.^[^ ^1^ ^]^ Boivin et al. state that “academic organizations have broadly failed to take the steps necessary to dismantle entrenched structural biases” and highlight the urgent need for genuine systemic transformation. In addition, Boivin et al. discuss how higher education and research institutions (HERIs) and governments should address gender‐based bias and harassment and ensure adequate support of female academics. The current comment highlights that we also need to consider the inherent and systematic inequity in academia including funding models, the underlying causes of inequity at individual HERIs as well as countries, and the trickle‐down impacts of equity in senior management positions.

## 2 How to Achieve Equity in a System that is Based on Inequity?

As the “She Figures”^[^ ^2^ ^]^ from the Directorate‐General for Research and Innovation show, the leaking pipeline is even more pronounced in the Science, Technology, Engineering, Mathematics, and Medicine (STEMM) field than in any other fields of academia. One reason for this pronounced leaking pipeline is that STEMM research is expensive because of the heavy and constantly evolving need for state‐of‐the‐art infrastructure and other resources (e.g., for laboratories, equipment, chemicals, skilled staff, etc.) that require internal investment and/or external co‐investment. However, HERIs do not receive a sufficient budget to adequately fund all academic staff—both male and female—at all career stages which subsequently leads to inequity among the academics competing for internal support of their research, i.e., some academics receive sufficient funding to advance their career while others unfortunately may never realize their full potential without sufficient support despite being capable researchers.

Therefore, inadequate provision of internal financial and infrastructure support leads to the challenge of striving for equity in a system that is fundamentally based on inequity. Enhanced transparency of how internal funding and infrastructure support is distributed amongst all academics might help to reduce bias and unhealthy competition. It is well known that bias perpetuates where transparency is lacking. If internal funding is limited, which is often the case, open calls for applications and transparent criteria and justifications should be an industry standard, as it is when academics apply for external funding.

In addition, another measure to improve equity is to report on the funding that each academic has received from their HERI—including funding received outside of open calls—and to account for this transparent investment with appropriate key performance indicators. Such an approach might help put the recipient's academic output into perspective, i.e., explain research outputs relative to opportunity. This measure is important to achieve equity because if a HERI is investing heavily in one research group and not others, comparison of academic outputs, which occurs during performance reviews and in promotion cases, should account for the clear benefits received by this higher investment and should be assessed accordingly.

## 3 Establishing the Underlying Causes of Inequity

Improving gender equity needs to be based on a better understanding of the reasons why women at early, mid‐, and advanced career stages face sexism and inequity. The underlying reasons likely differ from nation to nation and at institutional levels—even at faculty and school levels. A deep dive to unlock the causes of inequity is needed. However, this deep dive can be hindered by inadequate support from Academic Leaders or by Human Resource Managers who are focused only on data and privacy protection. We urgently need to stop measuring equity on basic statistics alone—sexism is complex; therefore, the focus must be on individuals, teams, and the specific situation at the HERI and school level. Statistics shine a light on the problem of inequity and barely on the genuine underlying causes. A deep dive into the causes of inequity at the HERI/faculty/school would also support the development of a bespoke and enforceable gender equity plan that would better address sexism and inequity at the HERI/faculty/school. As Boivin et al. state, “a broad, system‐level transformation is needed” but this transformation must be paired with an understanding of challenges at the individual HERI. The nitty gritty day‐to‐day details of inequity matter greatly.

## 4 Percentage of Women in Senior Management Positions as Key Performance Indicator

In addition, Boivin et al. stress the importance to mandate a percentage of women participation for decision‐making bodies (e.g., 40–50%) and for senior management to achieve gender balance. HERIs tend to report the (improved) percentage and number of women in senior management positions and decision‐making bodies as an indicator of equity at their institution. It goes without saying that it is a positive development when more women have positions in senior management, but it also should be noted that it is much easier to achieve parity in a small group of women and men than across the larger workforce. Even worse, this type of goalsetting can mask real issues and be a barrier for women experiencing inequities to find solutions to the issues they are being faced with. Therefore, a key performance indicator mandating proportions of women in senior management positions should be linked with evidence showing improvements in equity at all academic levels and at individual faculties and schools to demonstrate the true impact of equity in senior management positions.

## 5 Conclusion

Academia must lead the way in addressing sexism. Enhanced transparency and accountability as well as solid and well‐researched knowledge of the underlying causes of sexism in academia are urgently needed. As we educate the next generation of leaders across many impactful and influential professions, we must set the expectation in our own hallways to benefit our colleagues but also our students and wider communities that sexism must end.

## Conflict of Interest

The authors declare no conflict of interest.
